# Mild hypoglycemia is independently associated with increased mortality in the critically ill

**DOI:** 10.1186/cc9817

**Published:** 2011-03-11

**Authors:** J Krinsley, MS Schultz, P Spronk, R Harmsen, F Van Braam Houckgeest, J Van der Sluijs, C Melot, JC Preiser

**Affiliations:** 1 Stamford Hospital, Stamford, CT, USA; 2 Academic Medical Center, Amsterdam, the Netherlands; 3 Tergooi Hospitals, Blaricum, the Netherlands; 4 Medical Center Haaglanden, The Hague, the Netherlands; 5 Erasme University Hospital, Brussels, Belgium

## Introduction

Severe hypoglycemia (blood glucose level (BGL) <40 mg/dl) is independently associated with an increased risk of mortality in critically ill patients. The impact of milder hypoglycemia (BGL <70 mg/dl) on outcome is less clear.

## Methods

Prospectively collected data from two observational cohorts in the USA and in the Netherlands and from the prospective GLUCONTROL trial were analyzed. Hospital mortality was the primary endpoint.

## Results

We analyzed data from 3,262 patients admitted to Stamford Hospital (ST), 2,063 patients admitted to three institutions in the Netherlands (NL; loose glycemic protocol (L, *n *= 1,098) and strict glycemic protocol (S, *n *= 965)) and 914 patients who participated in the GLUCONTROL trial (GL; control arm (C, *n *= 460) and intensive insulin therapy arm (IIT, *n *= 454)). The percentage of patients with hypoglycemia varied widely among the different cohorts. Patients with hypoglycemia experienced higher mortality than did those without hypoglycemia within each subgroup (*P *< 0.0001 for all comparisons), even after stratification by severity of illness or diabetic status. Multivariable logistic regression analysis revealed that hypoglycemia had a greater impact on the mortality of surgical patients than of medical patients. The impact of hypoglycemia on mortality occurred independently of mean glucose level during ICU stay or glycemic variability.

## Conclusions

Even a single episode of mild hypoglycemia was associated with a significantly increased risk of mortality in heterogeneous cohorts of critically ill patients, independently of severity of illness, diabetic status, diagnostic category and glycemic variability.

